# Story telling in bilingual Urdu−Cantonese ethnic minority children: Macrostructure and its relation to microstructural linguistic skills

**DOI:** 10.3389/fpsyg.2023.924056

**Published:** 2023-02-27

**Authors:** Angel Chan, Sarah Chen, Saboor Hamdani, Bernard Tse, Kelly Cheng

**Affiliations:** ^1^Department of Chinese and Bilingual Studies, The Hong Kong Polytechnic University, Kowloon, Hong Kong SAR, China; ^2^Speech Therapy Unit, The Hong Kong Polytechnic University, Hong Kong, Hong Kong SAR, China; ^3^The Hong Kong Polytechnic University – Peking University Research Centre on Chinese Linguistics, Hong Kong, Hong Kong SAR, China; ^4^Research Centre for Language, Cognition, and Neuroscience, The Hong Kong Polytechnic University, Hong Kong, Hong Kong SAR, China

**Keywords:** bilingual ethnic minority children, Cantonese, macrostructure, narrative, Urdu

## Abstract

**Introduction:**

The ability to produce a well-structured, coherent and informative narrative requires the integration of lexical and grammatical skills at different levels of complexity. Investigating how narrative macrostructure competence is predicted by microstructural linguistic skills is conceptually enlightening; yet there have been very few, if any, studies documenting the associations between macrostructure and microstructure in both languages of the same bilinguals. In this paper we attempt to address this research gap and report on the first empirical study of Urdu-Cantonese bilingual children’s narrative abilities, bringing in data from a new language pair that is currently understudied.

**Methods:**

Twenty-four bilinguals (mean age = 9.17 years) acquiring Urdu as first, family and heritage minority language, and Cantonese as second, school and majority language were assessed *via* Multilingual Assessment Instrument for Narratives (MAIN). We examined these children’s macrostructural competence and its relations to microstructural skills in both languages (Urdu and Cantonese). Three macrostructure components were scored as response variables: Story Structure (SS), Story Complexity (SC), Internal State Terms (IST). Four microstructural measures were scored as predictor variables: number of different words (NDW), mean length of Communication Units (MLCU), proportion of grammatical Communication Units (Gproportion), proportion of correct connectives linking the major episodic elements (Cproportion).

**Results:**

In regression analyses, NDW emerged consistently as a positive predictor of SS, SC and IST in both languages. MLCU and NDW were positive predictors of SS in the stronger L1, but NDW was the only positive predictor of SS in L2. By contrast, NDW and an index of syntactic competence (MLCU in L1, but Cproportion in L2) were significant or close-to-significant positive predictors of SC in both languages. NDW was the only positive predictor of IST in both languages. These findings suggested that the relationships between narrative macrostructure and specific microstructural abilities could manifest both similarly and differently between L1 and L2.

**Discussion:**

We discuss the findings by considering the unique nature of each macrostructure component and how each component might be related to specific microstructural linguistic skills. We suggest directions for further research and discuss how the current findings bring deeper implications for educators and clinicians in assessment, pedagogy, and intervention.

## 1. Introduction

Children’s linguistic competence in narrative production can be analyzed at two levels: macrostructure and microstructure. Macrostructure refers to a higher-order global organization of a story such as episodic structure and story grammar components (Heilmann et al., 2010). Microstructure involves more local level of language use and a more language-specific analysis of the internal linguistic structure such as lexical items, morphosyntax and connectives used in constructing a coherent narrative production (Gagarina et al., 2016). Although it has been shown that macrostructure and microstructure represent two distinct areas underlying narrative competence, they are not mutually exclusive (Liles et al., 1995). Given that the ability to produce a well-structured, coherent and informative narrative requires the integration of lexical and grammatical skills at different levels of complexity, examining the associations between narrative macrostructure and microstructural linguistic skills is conceptually illuminating. This study aims to investigate how macrostructural competence is predicted by microstructural skills in both languages of a group of bilingual ethnic minority children.

### 1.1. Analysis of macrostructure and microstructure

There can be more than one way of coding story macrostructure depending on the framework, e.g., Applebee (1978)’s six-levels framework, High-point analysis (Labov, 1972), and episodic analysis (Stein and Glenn, 1979). The commonly used episodic analysis, also the framework adopted in this study, analyzes a story based on story grammar, where story grammar elements/components (e.g., setting, initiating event, internal response, internal plan, attempt, consequence and reaction) constitute the episodic structures of a story. The terminology regarding narrative macrostructure is highly variable in the literature. Studies have used terminologies such as story content, event content, story structure, and story complexity. Due to these variations, we discuss the core concept of macrostructure below, to help readers relate the current study to the earlier studies.

One major dimension is the content structure of a story. Under an episodic analysis, this dimension identifies the macrostructure of a story by evaluating the presence of story grammar elements/components. Because the intentions and events represented by these story grammar elements/components involve logical temporal and causal relationships, being able to verbalize more of these story grammar elements/components would contribute to the coherence and richness of relevant content of a story. It therefore has a quantitative dimension on one hand (counting the number of story grammar elements present), while also contributes to the quality of a story (in terms of richness and coherence of story content) on the other hand.

The second dimension is to consider the complexity of a story concept. This notion is related to how a good story is defined. For instance, Stein and Glenn (1979) argued that a goal-directed action is the necessary basis for a minimal definition of a story. A good story has to make reference to the following dimensions of goal-based action: (i) an animate protagonist that can initiate intentional action, (ii) an explicit statement of the goal or desire of the protagonist (the story grammar component “Goal”), (iii) the overt action(s) performed to serve the protagonist’s goal (the story grammar component “Attempt”), and (iv) the outcome(s) as a consequence of the goal being attained or not attained (the story grammar component “Outcome.”) Goal-Attempt-Outcome are therefore identified as critical components or dimensions of goal-directed action that form a complete episode. Following this reasoning, Stein (1988) and Westby (2005) constructed decision trees that incorporated these concepts and showed how “a systematic increase in the number of dimensions of a goal-directed action sequence increases the complexity of a story concept” (Stein and Albro, 1997, p: 8). This dimension considers how well Goal-Attempt-Outcome is expressed according to these decision trees. It indicates at which level the child’s narrative macrostructure is according to the different levels of structural complexity: (a) are there complete episodes, which include all three Goal-Attempt-Outcome statements; (b) are there abbreviated or incomplete episodes, which include Goal, but lack a complete Goal-Attempt-Outcome structure (i.e., Goal, Goal-Attempt, Goal-Outcome); (c) are there only action or reaction sequences, which do not include Goal (i.e., Attempt-Outcome); and (d) are there are only isolated descriptions (i.e., only Attempt or Outcome statements) or statements reflecting none of the episodic components. Under these considerations, stories can systematically increase in their complexity, with (d) corresponding to the lowest level of complexity, and (a) the highest level of complexity.

The third dimension is to consider the use of internal state terms (IST) to explicitly refer to a character’s internal states in a story. IST overlap with terminologies such as mental state language (Bartsch and Wellman, 1995), internal states (Miller and Aloise, 1989), evaluations and inferences (Burns et al., 2012). They provide information about a child’s understanding of a character being a mental being having intentionality, goals, mental states, and feelings. They also provide information of a child’s understanding of the goals and intentions of characters as a child conceives a character’s actions as goal-directed. As such, IST not only draw upon linguistic abilities to verbalize knowledge about intentional actions and mental states of characters, but also theory of mind abilities as a child conceives a character’s internal states (see also study two of Reilly et al., 2004 for a socio-cognitive perspective). In story-telling, IST are often linked to story grammar elements such as goals, initiating events, and reactions at the macrostructural level, as a child attempts to structure an episode to include reference to an initiating event that may involve the internal state of a character, which triggers an intentional goal of a character that leads to a goal-directed attempt, which in turn leads an outcome as a consequence of the attempt, and then a character’s reaction as a result of the outcome. However, IST are also linked to narrative microstructure, because they require semantic skills to use the appropriate and diverse lexical items to verbalize the internal states, and syntactic skills as IST often involve metalinguistic (e.g., say, ask, etc.) and metacognitive (e.g., decide, believe, etc.) verbs that occur in complex syntactic structures. As such, IST are not always included as a narrative macrostructural index in the literature (e.g., Altman et al., 2016). Studies such as Silliman et al. (2002) considered IST as microstructure elements. Unlike the first two dimensions that consider primarily the episodic structure of a story, IST are closely related to linguistic measures due to their unique close connections to microstructure in addition to macrostructure. Their acquisition is therefore relatively more dependent on language-specific experiences. Since bilingual children may differ in the acquisition of mental terms between the two languages (Silliman et al., 2002; Altman et al., 2016), it is possible to find different degrees of association with linguistic measures in the two languages.

There are also variations between earlier studies in terms of how macrostructure was assessed methodologically. Regarding story content, although story grammars are often used, macrostructure can also be coded differently in terms of measures of main ideas (Bishop and Donlan, 2005), events (O’Neill et al., 2004), information units (Renfrew, 1997), or plot structure (Berman and Slobin, 1994), with a common aim of assessing the amount of relevant information in a story for these latter analyses. For instance, Mäkinen et al. (2014) assessed macrostructural competence by evaluating the amount of relevant information used in a narrative and used the term “event content” to refer to the dimension of story content, although their information units are not entirely identical to story grammar elements. In another study by Karlsen et al. (2016), macrostructure was coded based on the presence of eight plot elements, although they overlap with but are not entirely the same as the conventional story grammar elements. Even when story grammar elements are used as the unit of relevant informational content, there are also methodological variations between studies in terms of how they scored story grammar. For instance, Altman et al. (2016) assessed macrostructure using two parameters. One parameter involves the story content counting only Goal, Attempt and Outcome expressed but not the other story grammar elements. The second parameter concerns the complexity of the narrative in terms of the Goal-Attempt-Outcome episodic elements, where Attempt/Attempt-Outcome sequences received 1 point, incomplete episodes like Goal/Goal-Attempt/Goal-Outcome received 2 points, and complete Goal-Attempt-Outcome received 3 points. Bonifacci et al. (2018) also had two macrostructural parameters, but the scoring methods were different. The first parameter was termed number of macro-structural elements, counting the presence of a wider set of macro-structural elements (Goal, Attempt, Outcome, Mental States, Setting). The second parameter was termed level of macro-structural complexity. Four levels of scores ranging from low to high were identified (0, 1, 2, 3) corresponding to absence, low, medium and high complexity levels, respectively. Specifically, absence refers to absence of at least one Attempt and one Outcome, low refers to presence of both Attempt and Outcome, without verbalizing Goal, medium refers to presence of both Goal and Attempt or both Goal and Outcome as incomplete episodes, and high refers to presence of all three core components Goal-Attempt-Outcome in a complete episode. One unwanted consequence of these methodological differences is that they make it harder to assess the extent of which differences in findings between studies could be attributable to the differences in the methodology used. More preferable would be to make use of a common set of assessment materials and methods that are applicable cross-linguistically and cross-culturally, allowing one to draw comparisons across languages, cultures, and acquisition contexts with more stringent methodological controls (see Multilingual Assessment Instrument for Narratives under Method).

Microstructure, on the other hand, targets the narrator’s ability in using the target language to construct a coherent narrative. Microstructure measures typically assess competency in the following dimensions when constructing a narrative: productivity (or story length) and lexis, syntactic complexity, grammaticality, and discourse cohesion. Higher microstructural competence is therefore characterized by a person’s ability to use diverse vocabulary, syntactically complex and grammatically well-formed utterances, and greater discourse cohesion to construct a longer narrative. Since microstructure features target language-specific proficiency, they are subject to more variations between languages and between bilinguals and monolinguals, compared to macrostructure (Altman et al., 2016; Gagarina et al., 2016; Rodina, 2017). Due to space constraints, below we introduce those measures that have been commonly examined in narrative studies, particularly those that will be targeted in the current study.

Story length and lexis are often measured by the total number of clauses or Communication Units, total number of words with and without mazes, and the Number of Different Words (NDW). NDW represents the different types of word tokens used in a language sample and has been frequently examined in microstructure. Studies have reported that NDW is a sensitive developmental measure in bilingual acquisition (Uccelli and Paéz, 2007) and a sensitive measure to differentiate between children with and without language disorders in both monolinguals (Auza et al., 2018; Torng and Sah, 2020) and bilinguals (Altman et al., 2016; Gagarina et al., 2019c). While NDW can be seen as a measure of productivity (Justice et al., 2006; Mäkinen et al., 2014), it can be seen as a measure of semantic diversity in other studies (Westerveld and Gillon, 2010; Westerveld and Roberts, 2017), and many others including the current study see it also as a measure of lexical diversity (e.g., Altman et al., 2016; Auza et al., 2018).

Syntactic complexity can be indexed by different measures, for instance, Mean Length of Utterance (MLU), Mean Length of Terminable Units (MLTU), and Mean Length of Communication Units (MLCU). They are computed by the total number of word tokens without mazes divided by the number of the structural units selected, where the base structural unit could be an utterance (for MLU), a terminable unit (for MLTU), or a communication unit (for MLCU). The rationale is that a higher level of syntactic complexity is often indexed by a longer mean length (in words, sometimes in morphemes) of a structural unit in a language sample, especially for younger children. Among these three options, the current study, like others (e.g., Mäkinen et al., 2014; Altman et al., 2016), chose MLCU to facilitate more direct comparisons of results with other research groups. In MLCU, communication unit, defined as an independent clause with its modifiers (Loban, 1976), is taken as the base structural unit. There are also other indices of syntactic complexity, e.g., proportion of subordinating/coordinating constructions, but are beyond the scope of the current study (see Gagarina et al., 2015 for details).

Grammaticality can also be indexed by different measures, for instance, proportion of grammatically well-formed error-free utterance (Bedore et al., 2010; Eisenberg and Guo, 2013), proportion of grammatical Terminable Units (Zwitserlood et al., 2015), and proportion of grammatical Communication Units (Fiestas and Peña, 2004). They are computed by the number of error-free structural units divided by the total number of the structural units, where the base structural unit could be an utterance, a terminable unit, or a communication unit. The rationale is that a higher level of grammatical competence is indexed by a higher proportion of grammatical error-free structural units in a language sample. Among these options, the current study, like others (Fiestas and Peña, 2004), chose proportion of grammatical Communication Units (Gproportion) to facilitate more direct comparisons of results with other research groups. There are also other measures that focused instead on errorful (not error-free) units, e.g., percentage of ungrammatical clauses or sentences (Auza et al., 2018; Sheng et al., 2020), addressing grammatical competence from the reverse side.

Discourse cohesion is defined as “a semantic relation between an element in the text and some other element that is crucial to the interpretation of it” (see the seminal work by Halliday and Hasan, 1976, p: 8). The relation is marked by language-specific devices including conjunctions/connectives, reference, substitution, ellipsis and lexis which contribute to the cohesion of a text. Discourse cohesion has been reported to be a vulnerable domain in L2 acquisition and children with language disorders (Liles et al., 1995; Kupersmitt et al., 2014). Among the various candidate measures of cohesion, the current study focused on the proportion of correctly used connectives linking the major episodic macrostructure components Goal, Attempt, Outcome (Cproportion, see 2.4 under Method for computations). Cproportion was chosen because it captures how the more global macrostructures interact with the more local microstructures in discourse structuring to produce a coherent narrative—a measure that is closely related to the theme of this paper.

### 1.2. Associations between microstructure and macrostructure

The associations between microstructural and macrostructural abilities in narrative production have been examined in the literature. For instance, Stein and Albro (1997) reported that the longest stories, measured by the number of clauses as an index of productivity at the microstructural level, were also structurally the best developed goal-based stories at the macrostructural level in English-speaking children’s narrative production. Soodla and Kikas (2011) examined the relationships between macro- and micro- structural measures in Estonian-speaking children. With the quantity of story information units used as the macro-structure level variable, they reported a high and significant positive correlation between story grammar scores and story length (indexed by number of words), a weak but significant positive correlation between story grammar scores and mean length of communication units, and also a weak but significant negative correlation between story grammar scores and the ratio of grammatical errors (as an index of grammaticality). They advocated that “although macrostructure and microstructure are two distinct underlying areas of narrative competence (Liles et al., 1995), children’s performances at both levels are significantly associated and should be taken into consideration in narrative assessment” (Soodla and Kikas, 2011, pp: 231–232). Fernandez (2013) studied Spanish-speaking children and reported that second-order theory of mind scores and number of clauses in narrative production (as a measure of linguistic productivity and complexity) significantly predicted pragmatic language skills, where pragmatic language skill is an aggregate score involving not only the use of internal state terms and story grammar elements but also other measures such as use of performed evaluation devices and connectives in narratives. Mäkinen et al. (2014) studied Finnish-speaking children and reported that the number of different words (but not the number of communication units) predicted event content, at the macrostructural level, of their narrative production.

In studies involving bilingual children, Karlsen et al. (2016) examined predictors of narrative production in first-graders learning L2 Norwegian. Results showed that nonverbal cognitive abilities and home literacy support (indexed by number of children’s books at home) predicted story macrostructure; while micro-aspects of narrative production were best predicted by L2 linguistic skills (vocabulary and grammar), home literacy support (indexed by number of children’s books at home) and time spent in kindergarten. The study focused only on L2 and did not examine the associations in both L1 and L2 of these bilinguals. More recently, Bonifacci et al. (2018) examined the relationship between micro- and macro- structural competence in the narrative production of monolingual L1 and bilingual L2 Italian-speaking children. Regression analyses showed that MLU was a significant positive predictor of the number of macro-structural elements expressed in monolingual L1 Italian. The model was not significant for the bilingual L2 Italian group. Based on these findings the authors suggested that in monolinguals, narrative macrostructural competence is influenced by the syntactic complexity achieved in the target language; while for bilinguals macrostructural story quality appears to be scarcely influenced by the linguistic structure of the narrative production in L2. This study focused on comparing monolingual L1 versus bilingual L2 Italian and did not examine comparisons of L1 versus L2 in the same bilinguals.

To date there has been little information documenting the associations between macrostructure and microstructure in both languages of the same bilinguals. We do not know much about how the nature of relationship(s) between narrative macrostructure and microstructure might be similar or dissimilar between a bilingual child’s L1 versus L2, or between the dominant versus weaker language. This investigation is conceptually important to the field, as it could contribute to our understanding of whether the relationships between macrostructure and microstructure competencies are affected by bilingual factors such as L1/L2 status, dominance patterns between the two languages, language proficiency of the two languages, typological distance between the two languages, and cross-linguistic influences between the two languages.

### 1.3. Current study

This study aims to add to the existing evidence based on the associations between narrative macrostructure and microstructure competence, in both L1 and L2 of the same bilingual children, bringing in data from a new language pair (Urdu-Cantonese) that is currently understudied. Urdu and Cantonese are typologically diverse languages with low typological proximity and little resemblance/overlap in form-function mappings between the two languages to facilitate positive transfer of L1 linguistic skills to L2. As such, similar patterns in macrostructure-microstructure relationships between two typologically distant languages could reflect the unique nature of particular macrostructure competencies. On the other hand, different patterns in macrostructure-microstructure relationships between two typologically distant languages could reflect the effect of bilingual factors such as L1/L2 status, dominance patterns between the two languages and/or language proficiency of the two languages. Investigating macrostructure-microstructure relationships in both languages of the same bilingual children offers a unique opportunity of a within-subjects design to examine the cross-linguistic manifestation of these possible relations and test these conceptual perspectives.

The study also capitalizes on the methodological and theoretical strengths of MAIN, using the newly adapted Urdu and Cantonese versions of MAIN to conduct dual language assessment (Gagarina et al., 2019a,b; Chan et al., 2020; Hamdani et al., 2020; Kan et al., 2020). Our research questions are:

1. How do the patterns of association between macrostructure and microstructure measures resemble and differ between these three macrostructure dimensions/components (story structure, structural complexity, and internal state terms)?
2. How do the patterns of association between macrostructure and microstructure measures resemble and differ between L1 and L2?

The current study features a group of bilingual ethnic minority children who acquire both languages in conditions of reduced input, a prominent acquisition challenge. These children acquire their heritage language (Urdu) as first and family language and acquire the majority and societal language (Cantonese) as a second or additional language when residing in Hong Kong. They mainly receive input in their first language at home, but not in society or school due to smaller number of speakers and the minority status of their heritage language. Moreover, these families often have restricted social contacts with native speakers of Cantonese, which means the amount of contact with the target language is also reduced. Lacking integration into the community and support from parents, many of whom do not speak Cantonese, these children also face the challenge of acquiring Cantonese under reduced input. They are also associated with lower SES family status (Huttenlocher et al., 2010), which ultimately may affect the quantity and quality of their language learning experiences, since in many studies higher family SES and parental (esp. maternal) level of education have been associated with a child’s good language development (e.g., Dollaghan et al., 1999; Armon-Lotem et al., 2011). Examining the relationships between macrostructure and microstructure in these children provide new evidence to consider how these relationships are manifested in a unique acquisition context where these children develop their narrative competence under generally reduced and disadvantaged input conditions in both languages.

## 2. Methods

### 2.1. Participants

Twenty-four (13 females) bilingual Urdu-Cantonese children aged between 6 and 12 years old (M = 9.17 years, SD = 1.68 years) attending local primary schools grades one to six in Hong Kong participated. A parental questionnaire was completed to obtain background information on children’s demographic data, developmental history and language environment. All participants were considered as typically-developing based on the following justifications: (i) no reported noticeable delay in major developmental milestones in L1, considering both the onset of first word and word-combination; (ii) no reported concerns regarding speech and language development from parents and teachers; and (iii) no suggestive evidence for intellectual disability based on their non-verbal reasoning performance assessed by Raven Progressive Matrices test (standard score, M = 91.5, SD = 12.2, Range = 73–125; Raven et al., 1996).

These children were born in Hong Kong, so their chronological age and length of residence is identical. They come from the Pakistani heritage community acquiring Urdu as their first, family and minority language since birth. They started to be exposed to Cantonese on a more regular and intensive basis since they started schooling around age 3 in local schools using Cantonese as the medium of instruction, acquiring Cantonese as their second, school and majority language.

### 2.2. Materials, tasks, and procedures

Oral narratives were elicited using Multilingual Assessment Instrument for Narratives (MAIN; Gagarina et al., 2019) adapted to Cantonese (Gagarina et al., 2019a; Chan et al., 2020) and Urdu (Gagarina et al., 2019b; Hamdani et al., 2020). Unlike other narrative assessment tools, MAIN is uniquely designed for dual language assessment in bilinguals. It contains four stories that are parallel in content and structure to assess macrostructure and microstructure abilities and allows systematic comparisons between the two languages of a bilingual child. Moreover, MAIN is cross-linguistically and cross-culturally robust, with over 80 language versions being used in research. The story scripts of these language versions follow the standardized adaptation process (Bohnacker and Gagarina, 2020) to ensure that macrostructural features are the same across languages, while microstructural features like number of words per story (+/−3), number of direct speech sentences are as similar as possible across stories and to the English version.

MAIN also has its theoretical appeal in studying narrative macrostructure. It incorporates ideas from story grammar theory (Mandler, 1979; Stein and Glenn, 1979), causal framework analysis (Trabasso and Nickels, 1992), and the binary story grammar decision tree (Westby, 2005) which consider not only the presence of story grammar elements, but also the causality involved between the main episodic components GAO, and the level of structural complexity and developmental level of narratives. Under a multi-dimensional approach in studying macrostructure, MAIN distinguishes 3 components of macrostructure: Story Structure (SS), Structural Complexity (SC) and Internal State Terms (IST). SS considers the story content organization in terms of counting the number of story grammar elements produced, aligning with the first dimension of evaluating the richness and coherence of the content structure a story. SC considers the complexity of combinations of the main components Goal-Attempt-Outcome in an episodic structure based on the binary decision tree (Westby, 2005), aligning with the second dimension of evaluating the level of structural complexity of a narrative. IST refer to words that express the internal states of a character generally referring to feelings and mental states such as intentions, thoughts, emotions, and reactions of characters in the story, aligning with the third dimension of evaluating the use of language to explicitly refer to the internal states of characters in a narrative production.

Each child completed two stories in Cantonese and another two in Urdu. The order of the language assessed was counterbalanced between participants, where half were assessed in Urdu first and in Cantonese second, while the other half in Cantonese first and in Urdu second. Following MAIN’s instructions (Gagarina et al., 2019a,b), the stories Cat and Dog were administered in different languages, and Baby-Birds and Baby-Goats were also administered in different languages. The stories assigned to a particular language were also counterbalanced between participants, allowing the four possible story combinations (Cat-Baby Birds, Cat-Baby Goats, Dog-Baby Birds, Dog-Baby Goats) to be used evenly in equal number of times in both L1 and L2 across children as a group (see “counterbalancing procedures for research purposes” in Gagarina et al., 2019a,b). Moreover, each story was assessed twice, once in telling and then in retelling. Specifically, in telling, the child had to generate and tell a story based on the pictures to the experimenter. Then, in retelling, the child would listen to a pre-recorded model story along with the pictures, and then be expected to retell the story.

### 2.3. Macrostructure measures

Three macrostructure dimensions/components: story structure, structural complexity, and internal state terms were scored as response variables in both languages.

*Story structure (SS).* All four stories began with a setting (i.e., time, place), followed by three short episodes, each consisting of an initiating event, Goal, Attempt, Outcome, and a reaction. Each story produced was scored in terms of the number of story grammar elements verbalized. Each element scored for 1 point. Maximum 17 points for each story.

*Structural complexity (SC)*. SC was measured using a 3-point weighting system adapted from Maviş et al. (2016). A sequence without Goal (i.e., Attempt-Outcome) would be given 1 point. An incomplete episode (single Goal, Goal-Attempt or Goal-Outcome) would be given 2 points. A complete episode (Goal-Attempt-Outcome) would be given 3 points. Maximum 9 points for each story.

*Internal state terms (IST).* The tokens of IST were counted following the MAIN manual. All instances of perceptual state terms (e.g., Cantonese: 睇; Urdu: سنا، دیکھا), physiological state terms (e.g., Cantonese: 肚餓; Urdu: پیاسا، بھوکا), consciousness terms (e.g., Cantonese: 瞓着; Urdu: جاگا، زندہ), emotion terms (e.g., Cantonese: 傷心; Urdu: اداس، خوش), mental verbs (e.g., Cantonese:決定; Urdu: سوچا، چاہتا), linguistic verbs or verbs of saying and telling (e.g., Cantonese: 講; Urdu: بلایا، چینخا) produced were counted in each story.

### 2.4. Microstructure measures

The following four measures were calculated for each story produced as predictor variables in both languages. Although measures of productivity such as total number of word tokens and number of communication units have been identified as having associations with macrostructure competence in the literature, they were not included in this study. This is because Poisson regression model adopted here (see section 2.5 for justifications) requires the measures to be independent as a pre-requisite. To ensure that the predictor variables are all independent, we kept MLCU but did not include the total number of word tokens and number of communication units because calculation of MLCU was derived from total number of word tokens divided by number of communication units.

*Number of different words (NDW).* NDW represents the number of different words without mazes, disregarding repeated word tokens. Since words are used in syntactic structures in narratives, NDW can be viewed as reflecting lexico-grammatical competence. NDW has been reported as having significant positive associations with macrostructure competence in Altman et al. (2016) and Mäkinen et al. (2014).

*Mean length of Communication Units (MLCU).* MLCU was computed by the total number of word tokens without mazes divided by the number of Communication Units. It is a typical measure of syntactic complexity and has been reported as having associations with macrostructure competence (Soodla and Kikas, 2011).

*Proportion of grammatical Communication units (Gproportion).* Gproportion, a measure of story grammaticality, was calculated by the number of grammatical Communication Units produced divided by the total number of Communication Units. It could be particularly interesting in a weaker L2 context when grammatical (in)competence may be sensitively captured by significantly fewer grammatical sentences. Grammaticality has been examined in Soodla and Kikas (2011), although they found only weak associations with macrostructure.

*Proportion of correctly used connectives linking the major episodic components (Cproportion).* Cproportion, a measure of narrative cohesion, was calculated by “the number of correctly used connectives divided by the total number of Goal-Attempt-Outcome (or any of the two, i.e., Goal-Attempt, Attempt-Outcome, Goal-Outcome) produced in a story sample.” Connectives including additive, causal, sequential and adversative connectives that were used to connect any of the two or all three main episodic components (i.e., Goal and Attempt in Goal-Attempt, Attempt and Outcome in Attempt-Outcome, Goal and Outcome in Goal-Outcome, or Goal and Attempt and Outcome in Goal-Attempt-Outcome were counted as long as they were used correctly). The number of sequences (Attempt-Outcome), incomplete episodes (Goal-Attempt, Goal-Outcome), and complete episodes (Goal-Attempt-Outcome) produced were included in the calculation of the total number of Goal-Attempt-Outcome. Liles et al. (1995) reported that their index of cohesion was moderately related to narrative macrostructure, suggesting that some aspects of cohesion may facilitate a higher-order level of story organization.

### 2.5. Transcription, scoring and data analysis

The narrative samples were transcribed by a native speaker of the respective language and then cross-checked by one more native speaker to ensure accuracy. Independent scoring of macrostructure and microstructure were carried out by two native speakers of the respective language who were student speech therapists (Cantonese) or research assistants (Urdu) with relevant training. Discrepancies were resolved through discussion with the first author. The Urdu scorings were cross-checked by one more native speaker who is a speech therapist from Pakistan doing her PhD in Hong Kong (third author).

Poisson regression models were chosen because count variables were involved, and they followed a Poisson distribution. A count variable is defined as a variable reflecting the number of occurrence of certain events and it takes on positive discrete values such as 0, 1 and 2 (Coxe et al., 2009). For example, since SS refers to number of story elements expressed, and IST refers to number of internal state terms expressed, they are considered as count variables. Using the standard ordinary least squares (OLS) regression can be potentially problematic because it usually requires the random errors to follow a normal distribution N(0,σ2; Meloun and Militký, 2001). If a count variable is used as an outcome variable in OLS regression, and when the mean of the variable is low, OLS regression models are likely to produce biased results (Gardner et al., 1995).

In the first round of analyses, the data were analyzed with each of the four microstructural measures [Number of Different Words (NDW), Mean Length of Communication Units (MLCU), Proportion of Grammatical Communication Units (Gproportion), Proportion of correctly used Connectives linking the major episodic components (CProportion), Age, Elicitation Mode (telling vs. retelling), Language, and the two-way interaction terms between Language and each of the other predictors as predictor variables, and each of Story Structure (SS), Story Complexity (SC) and Internal State Term (IST) scores as a response variable in a model (i.e., one model for one response variable)]. The interaction terms with Language allow us to identify whether the effect of a predictor variable on a response variable of macrostructural competence was uniform or not across languages. Since we identified several significant two-way interactions with Language, in the second follow-up round of analyses, we therefore ran the analyses separately within each language. In this follow-up round of analyses, we conducted two sets of analyses. One set was simple bivariate correlations between each predictor variable and each macrostructure outcome variable within each language. Spearman’s correlation coefficient was used for all the correlations except for Elicitation Mode, where point biserial correlation coefficient was used, as telling/retelling is a categorical variable. The second set of analyses were regression analyses within each language, which entered all predictor variables into a regression that would allow us to consider how a variable reflected its contribution, while taking into account the contribution of all other variables. As such, the predicted shared variance is distributed across all predictor variables. Specifically in these regression analyses, the data were analyzed with each of the four microstructural measures [Number of Different Words (NDW), Mean Length of Communication Units (MLCU)], Proportion of Grammatical Communication Units (Gproportion), Proportion of correctly used Connectives linking the major episodic components (CProportion), Age and the Elicitation Mode (telling vs. retelling) as predictor variables, and each of Story Structure (SS), Story Complexity (SC) and Internal State Term (IST) scores as a response variable in each model (i.e., one model for one response variable in a particular language).

The findings are considered significant with *p* values less than 0.05. Estimated rate ratio represents the expected value of increase (if the estimated coefficient of a variable is positive) or decrease (if the estimated coefficient of a variable is negative) of the assessed macrostructure dimension/component, if a participant were to increase a particular predictor variable by one unit, while holding all other variables in the model constant. For example, if the estimated rate ratio is 1.01 for a one-unit increase of a predictor variable [e.g., Number of Different Words (NDW)] in affecting scores of a response variable (e.g., Story Structure (SS)) and that the estimated coefficient is positive, this means that if the participants were to increase their NDW by one unit, their rate ratio for SS would be expected to increase by a factor of 1.01, while holding all other variables in the model constant. The higher the estimated rate ratio, the greater contribution the respective predictor variable has in the model.

## 3. Results

### 3.1. Language dominance

We also examined children’s narrative skills in both languages. This gives background knowledge on which language (Urdu vs. Cantonese) could be the dominant language. Table 1 shows each of the seven measures comparing Urdu versus Cantonese. The results were generated by fitting a Poisson regression model for each measure as the dependent variable and language (Urdu vs. Cantonese) as the independent variable to examine if there are any significant differences between languages.

**Table 1:** The bilingual children’s scores in Urdu versus Cantonese on the seven measures of macrostructure and microstructure abilities.

Nature of linguistic competence	Measures	Urdu *mean (SD) range*	Cantonese *mean (SD) range*	*z* value	*p* value
Macrostructure	SS	9.72 (2.49), 3–14	9.09 (2.79), 3–15	1.98	0.048*
	SC	4.29 (2.12), 0–9	3.92 (2.17), 0–9	1.82	0.068
	IST	6.40 (4.20), 0–24	7.38 (3.96), 1–21	−3.63	0.000***
Expressive lexical or lexico–grammatical	NDW	50.52 (14.07), 25–88	42.57 (14.38), 19–83	11.37	<0.0001***
Syntactic complexity	MLCU	7.75 (1.55), 3.35–13.89	7.41 (15.01), 3.42–12.23	1.19	0.234
Grammaticality	Gproportion	0.822 (0.128), 0.38–1	1.15 (0.351), 0.29–2	−3.26	0.001**
Discourse cohesion	Cproportion	0.367 (0.347), 0–2	0.328 (0.441), 0–2	0.644	0.52

*p < 0.05. **p < 0.01. ***p < 0.001; SS = Story Structure; SC = Story Complexity; IST = Internal State Terms; NDW = Number of Different Words; MLCU = Mean Length of Communication Units; Gproportion = Proportion of grammatical Communication Units; Cproportion = Proportion of correctly used connectives linking the major episodic components.

The following measures all consistently indicated that the Urdu scores were significantly or numerically higher than the Cantonese scores, suggesting that these children are largely dominant in their L1 Urdu: Story Structure (SS), Story Complexity (SC), Number of Different Words (NDW), Mean Length of Communication Units (MLCU), Proportion of Grammatical Communication Units (Gproportion), Proportion of correctly used Connectives linking the major episodic components (CProportion). This dominance pattern is consistent with information gathered from the parental questionnaires. Their parents reported in the questionnaires that these children spent more time in an Urdu-speaking environment than in a Cantonese-speaking environment. Specifically, when being asked “On average, how many % of hours per week does your child spend in each language environment (school + home + other environments all included) for Cantonese and for Urdu?,” 22 out of 24 parents expressed a higher percentage of weekly exposure in an Urdu-speaking environment than in a Cantonese-speaking environment, with only 2 out of 24 parents expressed an equal percentage of weekly exposure to Urdu and Cantonese. This dominance pattern is also consistent with parental evaluations of their children’s language proficiency of the two languages in the questionnaire. Specifically, when being asked “On a scale from 1 (poor) to 7 (excellent), please rate your child’s ability to understand/speak spoken Cantonese and Urdu,” 17 out of 24 parents gave a higher rating for Urdu than Cantonese in speaking and/or understanding, with only 7 out of 24 parents giving an equal rating for both languages in speaking and understanding. These 7 parents gave either one of the two highest ratings, i.e., a rating of 6 or 7, for both languages. It is also common that these ethnic minority parents are not proficient in Cantonese and therefore these families usually lack practices in supporting literacy in Chinese at home, although our parental questionnaire did not ask specifically about home practices in supporting literacy. There is some suggestive evidence from other responses in the questionnaire though. For instance, 20 out of 24 parents expressed that their child speaks more Urdu than Cantonese at home, suggesting lack of support for Cantonese from the family. The only measure for which Cantonese was stronger than Urdu was the children’s Internal State Term (IST) scores. It is possible that IST, compared to Story Structure (SS) and Story Complexity (SC), is more related to the child’s language-specific experience (see the introduction section on the unique linguistic nature of IST). This point will be elaborated further in the discussion section.

### 3.2. Macrostructure dimensions/components and their relationships with microstructure abilities

Tables 2, 3 present the simple bivariate correlation results in Urdu and Cantonese, respectively. The results showed a number of significant positive correlations between specific microstructural competencies such as Number of Different Words (NDW), Mean Length of Communication Units (MLCU), Age, Elicitation Mode and the outcome measures of macrostructural competencies in Story Structure (SS) and Story Complexity (SC), and Internal State Term (IST). Note that if the correlation efficient of Elicitation Mode is positive, it indicates that when the variable *x* takes on the value “1” (retelling), the outcome variable *y* tends to take on higher values compared to when the variable *x* takes on the value “0” (telling).

**Table 2:** Simple bivariate correlations between predictor variables and macrostructure outcome variables in Urdu.

Predictor/ macrostructure	NDW	MLCU	Gproportion	Cproportion	Elicitation mode (telling vs. retelling)	Age
SS	0.653***	0.547***	−0.014	−0.116	0.397***	0.261***
SC	0.517***	0.382***	0.003	−0.117	0.323***	0.212**
IST	0.663***	0.405***	−0.108	−0.065	0.501***	−0.018

**p < 0.01. ***p < 0.001; SS = Story Structure; SC = Story Complexity; IST = Internal State Terms; NDW = Number of Different Words; MLCU = Mean Length of Communication Units; Gproportion = Proportion of grammatical Communication Units; Cproportion = Proportion of correctly used connectives linking the major episodic components.

**Table 3:** Simple bivariate correlations between predictor variables and macrostructure outcome variables in Cantonese.

Predictor/ macrostructure	NDW	MLCU	Gproportion	Cproportion	Elicitation mode (telling vs. retelling)	Age
SS	0.711***	0.391***	0.046	0.015	0.422***	0.402***
SC	0.438***	0.253***	0.064	0.280***	0.269***	0.082
IST	0.694***	0.273***	0.070	−0.055	0.384***	0.216**

**p < 0.01. ***p < 0.001; SS = Story Structure; SC = Story Complexity; IST = Internal State Terms; NDW = Number of Different Words; MLCU = Mean Length of Communication Units; Gproportion = Proportion of grammatical Communication Units; Cproportion = Proportion of correctly used connectives linking the major episodic components.

We next focus on reporting the significant positive predictors measuring microstructural competences of each macrostructure dimension/component in Urdu (L1) and then Cantonese (L2) in the regression analyses, which allow us to consider how a variable reflected its contribution, while taking into account the contribution of all other variables, with the corresponding value of ps, z values and rate ratios presented in Tables 4, 5, respectively. We then comment on Age, Elicitation Mode, and the significant negative predictors (with *p* < 0.05 but negative z-value) collectively across both languages toward the end of this section. Data came from all stories told and retold in Urdu or Cantonese.

**Table 4:** Predictor variables and their relations to SS, SC and IST in Urdu.

Macrostructure	Predictor variables	*z* value	*p* value	rate ratio
SS	NDW	4.10	< 0.001***	1.01
	MLCU	2.24	0.025*	1.04
	GProportion	−0.73	0.47	0.87
	CProportion	−1.15	0.25	0.92
	Age	2.84	0.0045**	1.00
	Elicitation Mode (Telling)	−0.95	0.34	0.95
SC	NDW	4.23	<0.001***	1.01
	MLCU	1.71	0.088	1.05
	Gproportion	−1.65	0.10	0.64
	CProportion	−1.87	0.06	0.81
	Age	2.87	0.0041**	1.01
	Elicitation Mode (Telling)	−1.28	0.20	0.90
IST	NDW	9.61	<0.001***	1.03
	MLCU	−1.96	0.05	0.96
	GProportion	−6.21	<0.001***	0.26
	CProportion	−2.60	0.009**	0.78
	Age	−2.16	0.03*	1.00
	Elicitation Mode (Telling)	−5.17	<0.001***	0.70

*p < 0.05. **p < 0.01. ***p < 0.001; SS = Story Structure; SC = Story Complexity; IST = Internal State Terms; NDW = Number of Different Words; MLCU = Mean Length of Communication Units; Gproportion = Proportion of grammatical Communication Units; Cproportion = Proportion of correctly used connectives linking the major episodic components; Elicitation Mode (Telling) = Elicitation Mode with Telling as reference level.

**Table 5:** Predictor variables and their relations to SS, SC and IST in Cantonese.

Macrostructure	Predictor variables	*z* value	*p* value	rate ratio
SS	NDW	5.97	<0.001***	1.01
	MLCU	−0.13	0.90	0.99
	GProportion	−1.41	0.16	0.91
	CProportion	0.41	0.69	1.02
	Age	1.99	0.047*	1.00
	Elicitation Mode (Telling)	−4.14	<0.001***	0.82
SC	NDW	6.02	<0.001***	1.02
	MLCU	0.36	0.72	1.01
	GProportion	−0.25	0.81	0.97
	CProportion	4.07	<0.001***	1.38
	Age	−1.92	0.055	1.00
	Elicitation Mode (Telling)	−2.29	0.022*	0.84
IST	NDW	12.49	<0.001***	1.03
	MLCU	−1.95	0.052	0.96
	GProportion	−1.56	0.12	0.88
	CProportion	−0.79	0.43	0.95
	Age	−0.97	0.33	1.00
	Elicitation Mode (Telling)	−4.57	<0.001***	0.78

*p < 0.05. **p < 0.01. ***p < 0.001; SS = Story Structure; SC = Story Complexity; IST = Internal State Terms; NDW = Number of Different Words; MLCU = Mean Length of Communication Units; Gproportion = Proportion of grammatical Communication Units; Cproportion = Proportion of correctly used connectives linking the major episodic components; Elicitation Mode (Telling) = Elicitation Mode with Telling as reference level.

In Urdu, findings from Table 4 revealed both similarities and differences between the three macrostructure components in terms of their significant positive predictors. Story Structure (SS) and Story Complexity (SC), and Internal State Term (IST) were similar in terms of having Number of Different Words (NDW) emerged consistently as a highly significant positive predictor of all three macrostructure dimensions/components. In addition, Story Structure (SS) and Story Complexity (SC) were relatively more similar in terms of having Mean Length of Communication Units (MLCU) as a significant positive predictor (in SS) or a close-to-significant positive predictor (in SC), with MLCU having the highest rate ratio among all predictors in both SS and SC. IST, on the other hand, differed from SS and SC, as NDW emerged as its only significant positive predictor.

In Cantonese, findings from Table 5 also showed both similarities and differences between the three macrostructure dimensions/components in terms of the significant positive predictors. Story Structure (SS) and Internal State Term (IST) were similar in terms of having Number of Different Words (NDW) emerged as the only significant positive predictor among the four microstructural measures. In fact, NDW was consistently a highly significant positive predictor of all the three macrostructure dimensions/components. Story Complexity (SC), by contrast, differed from SS and IST, as it was related to an additional measure, Proportion of correctly used Connectives linking the major episodic components (CProportion), which had an even higher rate ratio than NDW (rate ratio of Cproportion = 1.38; rate ratio of NDW = 1.02).

Although it is reasonable to expect age-related improvements in macrostructural competence in both languages, when age was added as a predictor together with the other predictor variables in regression analyses, age did not emerge as the strongest (indexed by the highest rate ratio, or not even a significant positive) predictor relating to macrostructure competence in both languages. For instance, in L1 Urdu, although age was a significant positive predictor of Story Structure (SS) and Story Complexity (SC), its rate ratio was slightly lower than that of Mean Length of Communication Units (MLCU) and Number of Different Words (NDW). Moreover, was even a negative predictor (indicated by its negative z-value) of Internal State Term (IST). Similarly, in L2 Cantonese, although age was a significant positive predictor of SS, its rate ratio was slightly lower than that of NDW. Moreover, age was a non-significant predictor of IST, and was even a close-to-significant negative predictor (indicated by its negative z-value) of SC. This finding suggests that although age is often a cursory measure of length of exposure to a language (especially for L1 in acquisition studies), this relationship could be much less tight when L1 is a minority language and L2 a majority language in bilingual ethnic minority children. Rather, measures of quality and quantity of experience to each language are likely better candidate measures as predictors than age.

Regarding elicitation mode, as expected and consistent with previous studies (Pesco and Kay-Raining Bird, 2016), these children scored significantly higher in a number of macrostructure components in story retelling than telling (Internal State Term (IST) in Urdu: z = −5.17, *p* < 0.001; Story Structure (SS) in Cantonese: z = −4.14, *p* < 0.001; Story Complexity (SC) in Cantonese: z = −2.29, *p* = 0.022; Internal State Term (IST) in Cantonese: z = −4.57, *p* < 0.001), with the benefit of a prior script.

A minor remark is that there were also two reported significant negative predictors among the microstructural measures, namely Proportion of Grammatical Communication Units (Gproportion) and Proportion of correctly used Connectives linking the major episodic components (Cproportion) in predicting Internal State Term (IST) in Urdu, as indicated by their negative z values (see Table 4). Conceptually it is unclear why there is a negative relationship between grammaticality and IST and between discourse cohesion and IST in Urdu. However, it was observed in this dataset that somehow those participants scoring higher in Gproportion and Cproportion happened to score lower in IST in Urdu. Future investigations examining how other measures of grammaticality and discourse cohesion correlate with IST will allow one to further evaluate the robustness of these findings, before attempting to give an explanation.

### 3.3. Cross-linguistic comparisons in how microstructure abilities predict each macrostructure dimension/component

There were both cross-linguistic similarities and differences attested. Regarding similarities, Number of Different Words (NDW) was consistently a highly significant positive predictor of these children’s scores in Story Structure (SS) and Story Complexity (SC), and Internal State Term (IST) in both languages. Moreover, L1 and L2 were similar in IST in terms of having NDW emerged as the only significant positive predictor. Furthermore, L1 and L2 were similar in SC in terms of not only having NDW as a significant positive predictor but also having a grammatical skill-related microstructural measure as a positive predictor with a higher rate ratio, although the two languages also differed specifically with Mean Length of Communication Units (MLCU) emerged as the close-to-significant positive predictor in L1 Urdu, while Proportion of correctly used Connectives linking the major episodic components (CProportion) emerged as the significant positive predictor in L2 Cantonese. There was also a cross-linguistic difference attested in SS, as NDW was the only significant positive predictor emerged among all the four microstructural measures in L2 Cantonese, while in L1 Urdu MLCU and NDW emerged as important positive predictors.

## 4. Discussion

The current study examined whether and how macrostructure competence in each of the three components [Story Structure (SS) and Story Complexity (SC), and Internal State Term (IST)] and in both languages (L1 Urdu & L2 Cantonese) was (un)related to specific microstructural linguistic abilities in a group of bilingual ethnic minority children, where Urdu is stronger than Cantonese for many measures.

One robust finding is that Number of Different Words (NDW; rather than age) showed up consistently as a highly significant positive predictor of all three macrostructural dimensions/components in both languages. This result aligns with Mäkinen et al. (2014) reporting NDW as a significant positive predictor of macrostructure measures. Moreover, this result aligns with Altman et al. (2016) reporting significant positive correlations between NDW and their macrostructural complexity measure and between NDW and the use of mental state terms in the narrative production of English-Hebrew bilinguals. The current finding is conceptually justifiable. Macrostructure contributes to the overall meaning of a story and the overall meaning of a story is conveyed through the semantics of the diverse words deployed. In order to express different story grammar elements [Story Structure (SS)], verbalize and combine the core components Goal-Attempt-Outcome to form complete episodes [Story Complexity (SC)], and express internal state terms within a narrative production [Internal State Term (IST)], children have to deploy the relevant words productively in a narrative context as a basis to support verbalization of these three macrostructural dimensions. The convergent evidence from the three macrostructural dimensions/components and from both languages attested corroborates this argument.

There were also partially different profiles in the ways specific microstructural skills related to the three macrostructural dimensions/components in L1 and L2. The pattern of results for each macrostructural dimension/component would therefore be discussed next. Specifically, Story Structure (SS) was related jointly to Number of Different Words (NDW) and Mean Length of Communication Units (MLCU) in the stronger L1, but related only to NDW in the weaker L2. This finding suggests that macrostructural content (indexed by SS) in the stronger L1, characterized by significantly more informative narratives (indexed by the significantly higher SS scores in L1 than L2), was jointly influenced by lexico-grammatical and syntactic competence. In order to include more relevant information units (indexed by more story grammar elements) in a story, this dimension of macrostructural competence (indexed by SS) needs to be supported by not only the ability to use diverse relevant lexical items (indexed by NDW) but also requires the syntactic ability to combine relevant lexical items to form larger information units (indexed by MLCU). On the other hand, macrostructural content (indexed by SS) in these children’s weaker L2, characterized by significantly less informative narratives (indexed by significantly lower SS scores in L2 than L1), was related only to the ability to use diverse lexical items (indexed by NDW), and scarcely by the syntactic competence achieved in the target language. One may speculate that when it is about telling an informative story in a bilingual child’s weak L2, having adequate, diverse, and relevant vocabularies and being able to deploy them plays a more pivotal role. Our bilingual L1 results align with the monolingual L1 results in Bonifacci et al. (2018). They reported that Mean Length of Utterances (MLU) was a significant positive predictor of the number of macro-structural elements expressed in the narrative production of monolingual L1 Italian children but not in bilingual L2 Italian. The current findings are similar to theirs suggesting that in children’s L1, the quality of story macrostructure is influenced by the syntactic complexity (indexed by MLCU here, but indexed by MLU in Bonifacci et al., 2018) achieved in the same language. Whether this pattern of relationship is related to L1 status or proficiency in the dominant language is currently unclear, and will require further research to verify, for instance, comparing bilingual children with L1 as the weaker language.

Looking across both languages to compare Story Structure (SS) versus Story Complexity (SC), SC differed from SS in two respects. First, SC appears to be relatively more independent of general language proficiency. Unlike SS which showed significantly higher scores in L1 than L2 that aligned with the general language dominance pattern of these children, there was no significant difference in SC scores between L1 and L2 despite L1 being a stronger language in general. Conceptually, it is possible that the mental representation and knowledge of the core episodic structure Goal-Attempt-Outcome could be supported by transfer processes that are shared across the two languages, so SC is relatively more independent from linguistic proficiency in the target language, compared to SS. Second, SC was related not only to Number of Different Words (NDW) but jointly and even more related (indexed by a higher rate ratio) to a grammatical skill-related microstructural measure in both L1 and L2. This finding suggests that when one considers another macrostructural dimension in terms of the complexity of a story concept (indexed by SC), which taps into the ability to express and sequence the major components Goal-Attempt-Outcome as complete episodes in a narrative, this macrostructural competence needs to be supported by not only the ability to use diverse relevant vocabularies (indexed by NDW) but also requires some kind of syntactic competence in both L1 and L2. In L1, the syntactic competence to combine relevant words together to form larger information units (indexed by MLCU) was a close-to-significant predictor with the largest rate ratio. In L2, the syntactic competence to use cohesive devices (connectives) to connect the main episodic story grammar elements (indexed by Cproportion) emerged as a significant predictor with the largest rate ratio. This pattern of findings, manifested in both languages, might reflect the unique nature of SC. Recall SC measures children’s macrostructure competence in combining the core episodic components. Mean Length of Communication Units (MLCU) implicates children’s ability to combine and sequence relevant words to form longer information units; while Proportion of correctly used Connectives linking the major episodic components (CProportion) reflects children’s ability to use appropriate cohesive devices like connectives to connect the semantic relations between the main episodic elements Goal-Attempt-Outcome expressed in a story. Functionally, SC, MLCU and Cproportion, by nature, all draw upon children’s ability to connect and sequence some information/meaning units within a story. We speculate that this functional overlap observed between SC, MLCU and Cproportion might be relevant when attempting to make sense of the finding that SC was related to MLCU in children’s L1 Urdu and Cproportion in children’s L2 Cantonese, respectively. As for why MLCU showed up as the close-to-significant positive predictor in L1 but Cproportion showed up as the significant positive predictor in L2 is currently not entirely clear. Our findings showed that SC in a weak L2 context was unrelated to MLCU but more related to Cproportion in these bilinguals. Further research is needed to observe how robust this pattern of findings occurs in other bilinguals’ weaker L2.

Compared with Story Structure (SS) and Story Complexity (SC), Internal State Term (IST) is likely most related to language-specific experience given the unique linguistic nature of IST. We observed that IST scores were significantly higher in L2 Cantonese than L1 Urdu, despite L1 being the stronger language in general. Similar findings have been reported by Altman et al. (2016) who reported bilingual English(L1)-Hebrew(L2) children using more mental state terms in their L2, despite 10 of the 19 children being L1 dominant and 9 out of 19 being balanced bilinguals. They attributed this finding to language-specific experiences such as L2 school curriculum and the type of language input in school setting that frequently used mental verbs in L2. Similarly, we speculate that the higher IST scores in L2 Cantonese observed might be due to language-specific experiences during which these children experienced frequent use of ISTs in L2 local school curriculum and setting that uses Cantonese as the medium of instruction. The current findings also revealed cross-linguistic similarities in terms of Number of Different Words (NDW) being the only significant positive predictor of IST scores in both languages. It is conceptually predictable that the production of IST requires lexico-grammatical competence of deploying lexical items such as metalinguistic and metacognitive verbs and emotion words.

A further remark concerns the measure of grammaticality, indexed by Proportion of Grammatical Communication Units (Gproportion). Unlike Number of Different Words (NDW) which emerged consistently as a highly significant positive predictor of Story Structure (SS), Story Complexity (SC) and Internal State Term (IST) in both languages, in contrast, Gproportion consistently did not show up as a significant positive predictor of SS, SC, and IST in both languages. This finding aligns with Soodla and Kikas (2011), in the sense that their measure of grammaticality also showed only weak association with children’s macrostructural competence. One might speculate that the ability to produce grammatical communication units in narrative production does not appear to positively contribute to macrostructural competence in these bilinguals.

We make some further remarks about limitations of this study and suggestions for future research. There are likely large variabilities between participants within a relatively small sample size. Future studies with larger samples and a more restricted age range are needed to corroborate the current findings. Moreover, the aim was to examine the relationships between macrostructure and microstructure in these children, and as such we did not set out to include multiple age groups to examine age effects. Instead, since the children willing to participate in this study were of diverse age range, we included age as a predictor among other predictor variables in regression analyses to examine its relative contribution as a predictor of these children’s macrostructural competence. Future research could assess different age groups to examine whether the relationships between macrostructural and microstructural competence might vary at different ages. Furthermore, to delimit the scope of investigation, the current study only examined a language pair of L1 and L2 that are diverse with low typological proximity and little resemblance/overlap in form-function mappings between the two languages to facilitate positive transfer. As such, cross-linguistic similarities between L1 and L2 in the relationships between macrostructure and microstructure competencies are more likely reflecting the unique nature of particular macrostructure competencies, as we currently hypothesize, rather than likely due to L1 linguistic skills influencing those in L2. On the other hand, if the language pair involves typologically close languages, then similar patterns in macrostructure-microstructure relationships between the two languages could be due to similarities between L1 and L2 facilitating L1-to-L2 positive transfer of linguistic skills and the unique nature of particular macrostructure competencies. In this case, we may expect even more robust cross-linguistic similarities due to the synergistic effects of both factors. Future research could examine and compare more language pairs (typologically similar vs. typologically diverse) to test these conceptual perspectives in a natural within-participants paradigm within the same bilingual children.

Regarding application values, the current findings and their interpretations also give deeper implications for educators and clinicians in assessment, pedagogy, and intervention. Given that macrostructure is related to specific lexical and grammatical skills at the microstructural level, and certain microstructural skills may be more important than the others in relating to a dimension/component of macrostructural competence depending on L1/L2/proficiency status, one should not assess macrostructure and microstructure as if they are disjoint abilities, but should consider the nature of relationships between them. These perspectives also enlighten pedagogy/intervention, motivating one to discover more about how to foster a child’s lexical diversity along with building up her syntactic competence to support each of the three aspects of macrostructural competence in L1 versus L2. This line of inquiry is not restricted to the narrative genre and can be extended in future investigations to other academic discourse genres like exposition and argumentation, giving rise to a compositional construct of micro-properties of language that can predict competence in the macro-properties of language involving different genres of discourse in a child’s social-communicative and academic developments.

## 5. Conclusion

We reported on the first empirical study of Urdu-Cantonese bilingual children’s narrative abilities, bringing in data from a new and typologically distant language pair that is currently understudied. We examined macrostructural competence and its relation to specific microstructural linguistic skills in both languages of the same bilingual children, which, to our knowledge, has been under- or un- documented in the current published literature. We found that the significant predictor variables which were related to macrostructure competence were similar and partially different across SS, SC and IST. We discussed the findings by considering the unique nature of each macrostructure dimension/component and how each dimension/component might be supported by or related to specific microstructural linguistic skills. The take-home message is that while the cross-linguistic similarities observed provides convergent evidence in support of the unique nature of a particular macrostructure component, the cross-linguistic differences attested suggest that the possible relations between macrostructural and microstructural competence could vary between languages of a bilingual child that might be attributed to differences in language proficiency and/or L1/L2 status. Future studies assessing different groups of bilinguals with variations in their dominance profiles between L1 and L2 are necessary to tease these factors apart.

## Data availability statement

The original contributions presented in the study are included in the article/supplementary material, further inquiries can be directed to the corresponding author.

## Ethics statement

The studies involving human participants were reviewed and approved by the Institutional Review Board for human subjects ethics at the Hong Kong Polytechnic University (reference number: HSEARS20190813001-01). Written informed consent to participate in this study was provided by the participants’ legal guardian/next of kin.

## Author contributions

AC designed the study and interpreted the data in consultation with the other coauthors and recruited the participants, supervised native-speaker experimenters and research personnel in data collection, coding, reliability checks and analyses, and wrote a first draft of the paper. SC ran the statistical analyses. Subsequently all authors worked on refining and revising the text. All authors contributed to the article and approved the submitted version.

## Funding

This research was supported by a research grant (YW4G; P0014049; PI: AC), awarded by The Hong Kong Polytechnic University. SH is supported by a Hong Kong PhD Fellowship Scheme (HKPFS) awarded by the Hong Kong Research Grants Council.

## Conflict of interest

The authors declare that the research was conducted in the absence of any commercial or financial relationships that could be construed as a potential conflict of interest.

## Publisher’s note

All claims expressed in this article are solely those of the authors and do not necessarily represent those of their affiliated organizations, or those of the publisher, the editors and the reviewers. Any product that may be evaluated in this article, or claim that may be made by its manufacturer, is not guaranteed or endorsed by the publisher.
